# Reading after Confinement

**Published:** 1874-10

**Authors:** 


### READING AFTER CONFINEMENT.

Our attention has been called to the prevailing habit of ladies, especially those who are given to much reading during health, of attempting to somewhat relieve the tedium of the hours during which they are necessarily confined to their beds after parturition, by reading entertaining books and periodicals.

The use of the eyes under such circumstances is highly injurious, in many instances proving fatal to vision, as they are then very sensitive to light, so much so that the attending physician invariably recommends that the blinds be closed in order to render the light soft and agreeable to the eyes. To attempt to read under such circumstances, with inadequate light and in a recumbent position, with the book at varying distances from the eye, held with an unsteady hand, would cripple the strongest eyes. How much more would this result likely follow in the sensitive and enfeebled optics of the habitant of the lying-in-chamber.

One of the chief requisites of comfortable vision is, good light and that coming from the proper direction. In lying down when reading, more attention is given to the comfort of the body than that of the eyes, hence the danger of allowing the light to fall directly upon the face, or too fully reflected from the white pages of the book, directly into the eyes. Many ladies have their eyes seriously injured by attempting to read under the circumstances indicated. Reading should never be indulged in while lying down, and more particularly is this the case when the body is weakened through sickness.

### Healthy Dwelling Houses.

In a recent noteworthy and very elaborate paper on the proper mode of building houses so as to insure health, read by Dr. Hayward before the Liverpool Architectural Society, he enumerates various conditions essential to that purpose, the more important of which are a due exposure to fresh air and sunlight, positive freedom from damp, a large cubic space for air, and abundant means for the escape of foul and the admission of fresh air. Dr. Hayward argues that it is essential that the air should be warmed previous to admission, and that ventilation is the great and main necessity of house-building ; that whatever be left undone, this should be especially attended to. In regard to the temperature of the admitted air, he says that bedrooms are often very improperly constructed and arranged, so that in winter the sick occupant has to be in a current of air passing between the doorway and the fireplace, from twenty-eight to thirty-five degrees temperature, while that of his body is nearly one hundred. To these bedrooms, says Dr. Hayward, very many cases of consumption, bronchitis, and asthma may be traced ; furthermore, in fever cases much fresh air is required, and sometimes endeavor is made to obtain it even by opening the doors and windows, so that many typhus fever patients die of pneumonia, and many rheumatic fever cases also are prolonged and complicated. Drafts are equally pernicious in sitting-rooms, where persons may be roasted on one side and frozen on the other, resulting in neuralgia, rheumatism, colds, coughs, asthma, consumption, and a long train of similar ailments, the chilly lobby contributing materially to these results. Dr. Hayward urges the importance of a thorough reform in architectural construction in order to avoid these and other objections.—*Med. & Surg. Reporter.*

Cooking without Fire.—At Smoky Valley, Oregon, the people do not have the trouble of making a fire when they wish to get breakfast. They just walk out with their kettles, coffee-pots, and whatever else, and cook at the boiling spring. The water seems a great deal hotter than common boiling water, and all they need to do is to hang their kettle in it for a short time, and their food is nicely cooked. They are able even to bake in it. The bread is put into a tight saucepan, and lowered into the boiling flood for an hour or two, and then drawn up most exquisitely baked. It takes but a minute to cook eggs, or to make a pot of coffee or tea; but if there should happen to be a "slip between the cup and the lip," food would be gone beyond recovery.

